# Primitive Neuroectodermal Tumor/Ewing Sarcoma Presenting with Pulmonary Nodular Lesions

**DOI:** 10.1155/2015/957239

**Published:** 2015-01-29

**Authors:** Selvi Asker, Fuat Sayir, Gulay Bulut, Aysel Sunnetcioglu, Selami Ekin, Alpaslan Yavuz

**Affiliations:** ^1^Department of Chest Disease, Yuzuncu Yil University Faculty of Medicine, Van, Turkey; ^2^Department of Chest Surgery, Yuzuncu Yil University Faculty of Medicine, Van, Turkey; ^3^Department of Pathology, Yuzuncu Yil University Faculty of Medicine, Van, Turkey; ^4^Department of Radiology, Yuzuncu Yil University Faculty of Medicine, Van, Turkey

## Abstract

Primitive neuroectodermal tumors (PNETs) and Ewing sarcoma (EWS) belong to the same family of malignant, small, round cell neoplasms of soft tissue or bone origin. EWS-PNETs that arise in the lung parenchyma involvement are extremely rare in adults. A case of a 32-year-old male presenting with chest pain and diffuse pulmonary nodules on chest X-ray and diagnosed with Ewing sarcoma-PNETs will be presented here.

## 1. Introduction

Ewing sarcoma family of tumors (ESFT) are mainly aggressive sarcomas of bone and are also arising in soft tissues [1]. It is a group of small, blue, round cell neoplasms of neuroectodermal origin, which includes classical Ewing sarcoma, primitive neuroectodermal tumors, and Askin tumors of the chest wall [2]. Primitive neuroectodermal tumors (PNETs) and Ewing sarcoma (EWS) belong to the same family of malignant [3]. Common locations for EWS-PNETs include chest wall, pelvis, and extremities [3]. EWS-PNETs that arise in the lung parenchyma without chest wall involvement are extremely rare in adults. Primary PNETs of the lung are extremely rare, with fewer than 20 cases having been described in English literature to date [4].

75% of patients diagnosed with Ewing sarcoma are in the first 2 decades of their lives: the median age is 13 years and patients are rarely over 30 years of age [5]. ES has the ability to metastasize usually through hematogenous spread; most commonly, 38% metastasize to the lungs, 31% to bones (including the spinal column), and 11% to bone marrow [6]. According to past and recent studies, 15–40% of the patients already have metastasis at the time of diagnosis [7]. The rarity of primary pulmonary PNETs is also important to exclude the possibility of metastasis from a bone or soft tissue primary to the lung. Detailed examination by clinical and radiological means should be performed to rule out metastatic tumor from an extrapulmonary primary site [4]. We present the case of a 32-year-old case with EWS-PNETs of the lung that presented with chest pain to the pulmonology clinic, determined to have pulmonary nodules, and diagnosed with surgical removal of the nodule.

## 2. Case Presentation

**Figure 1:**
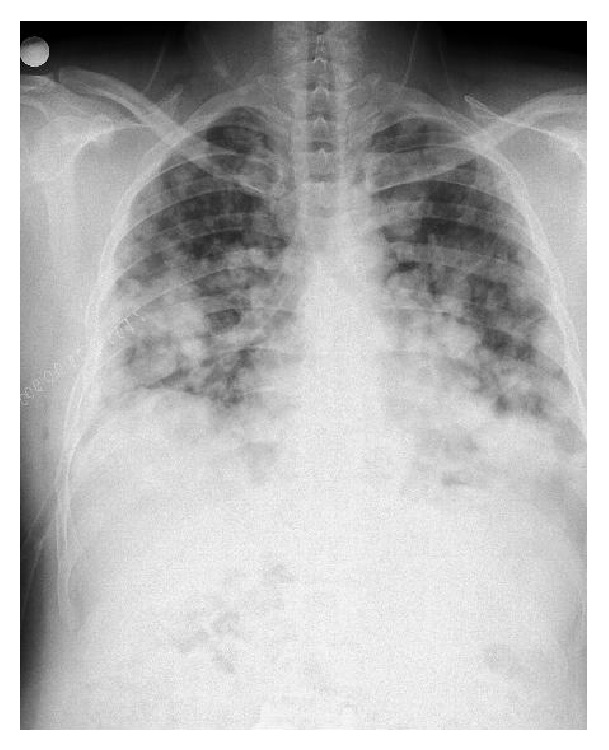
Nodular opacities varying in size present in all lung zones.

**Figure 2:**
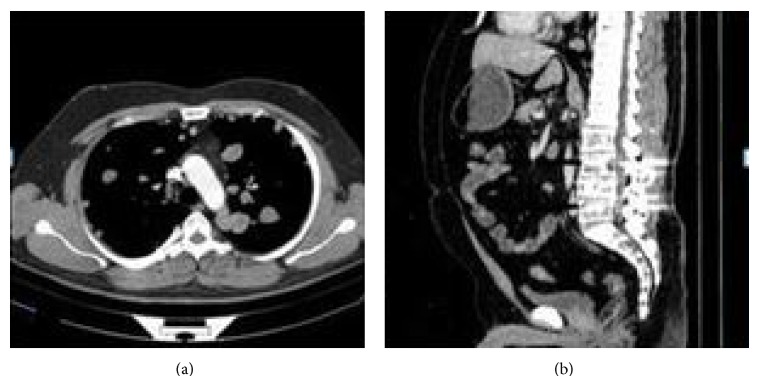
(a) Multiple parenchymal and subpleural tumors, lobulated contoured in some parts, located bilaterally in lungs, the largest measuring 5 × 4.5 cm on the left and 3.8 × 2.2 cm on the right. (b) Metal stabilizer screws extending from the L3–L5 vertebral peduncles to the corpuses, in addition to multiple lytic and sclerotic lesions on the L4 vertebra.

**Figure 3:**
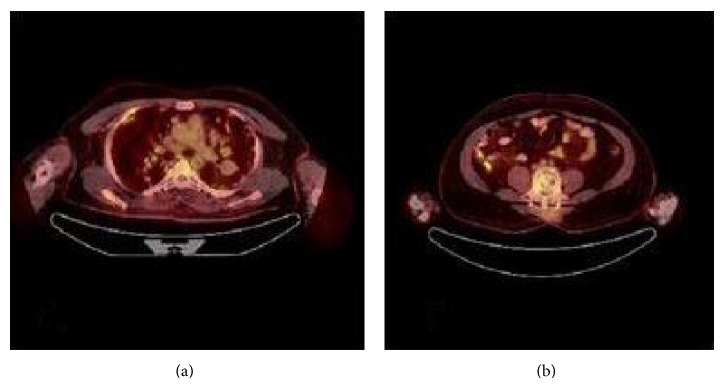
(a) Positron emission tomography with 18F-fluorodeoxyglucose (FDG) showed multiple round or oval lesions in the lung, (b) FDG uptake in midportion of L1-L2, L3 vertebral corpuses.

A 32-year-old male patient presented to the pulmonology department with the complaint of sleep apnea. Chest X-ray was performed due to the fact that the patient had a history of chest pain. Nodular opacities varying in size were observed in all lung zones on the chest X-rays (Figure 1). The findings of physical and laboratory examinations on admission were normal with no evidence of lymphadenopathy. His medical and family histories were unremarkable. We learnt that the patient had been operated 2 years ago and his vertebra had been fixated internally following a fall from a height, which had caused spondylolisthesis. Multiple parenchymal and subpleural tumors, with lobulated contours in some parts, located bilaterally in the lungs, the largest measuring 5 × 4.5 cm on the left and 3.8 × 2.2 cm on the right, were observed on the chest and abdominal computed tomography (CT). Metal stabilizer screws extending from the L3–L5 peduncles to the corpuses drew attention. Hypodense bone lesions, situated at the midportions of the vertebral corpuses of L1-L2-L3, the largest being 13 × 11 mm, drew attention (Figure 2). Department of Nuclear Medicine has stated that the patient's lesions on vertebrae can be related to the operation. Positron emission tomography with 18F-fluorodeoxyglucose (FDG) showed multiple round or oval lesions in the lung. The FDG uptake was increased in the lesions (maximal standard uptake value: 10,2) (Figure 3). The patient underwent bronchoscopy and a transbronchial lung biopsy, in addition to washing with cytology. The pathological diagnosis of the specimen was normal. Surgical resection was selected for the definitive diagnosis and treatment. Biopsy was obtained through wedge resection from the nodular lesions close to the pleura under general anesthesia.

**Figure 4:**
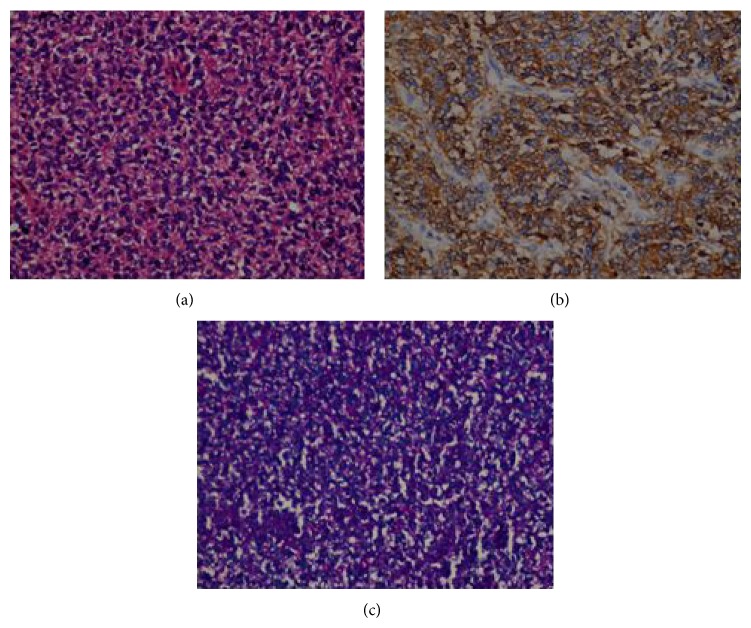
(a) Photomicrograph showing sheets of proliferating round cells, diffuse proliferation of cells with indistinct cytoplasm and small basophilic nuclei (HE ×400). (b) Strong and membranous CD99 immunoreactivity (CD99 immunohistochemical stain ×400). (c) shows abundant cytoplasmic glycogen in the cells of Ewing sarcoma with PAS stain. (PAS stain ×400).

Microscopically, the primitive cells were consisted solid sheets of cells divided irregular masses by fibrous strands. The tumour cells were small round/oval cells with scanty cytoplasm and uniform and uniform nuclei with dispersed chromatin. There were well developed vascular networks. Necrosis was common (Figure 4(a)). Immunohistochemically, the tumour cells were membrane positivity for CD99 (Figure 4(b)), vimentin and were negative for pan cytokeratin, desmin, synaptophysin, leukocyte common antigen, and myoglobin. PAS stain was positivity abundant cytoplasmic glycogen in the primitive cells (Figure 4(c)).

**Figure 5:**
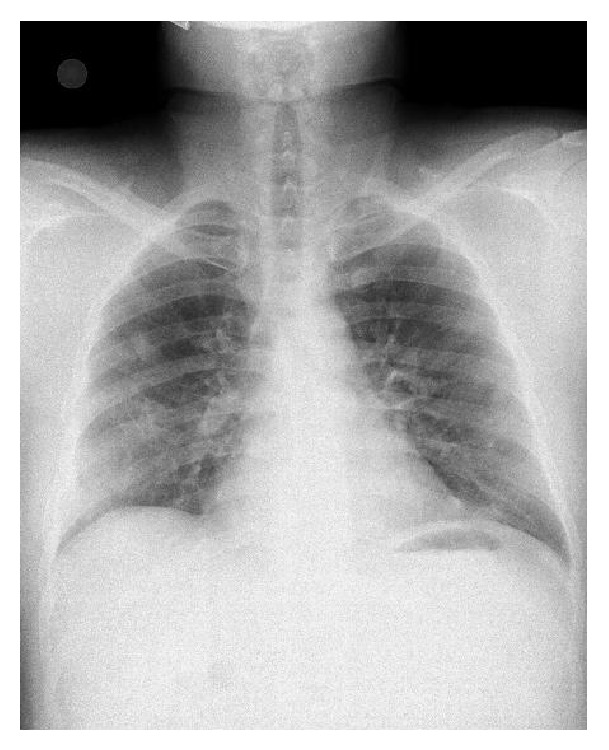
Regression of nodular lesions at the postoperative 2nd month follow-up chest X-ray following chemotherapy.

The patient was admitted to the medical oncology clinic for chemotherapy. At the outpatient clinic, the patient underwent 6 cycles of chemotherapy consisting of vincristine, oxorubicin, cyclophosphamide, iphosphamide, and etoposide. A decrease in the sizes of the nodular lesions or radiological disappearance was observed in the postoperative 2nd month follow-up chest X-rays following regular chemotherapy (Figure 5). After treatment no regression of lesions on vertebrae is observed. Treatment of the patient is continuing with a multidisciplinary approach with collaboration of neurosurgery, medical oncology, pulmonology, and orthopedics departments.

## 3. Discussion

Ewing sarcoma family of tumors are mainly aggressive sarcomas of bone and also arising in soft tissues, which share common features [1]. Our patient has nodular lung lesions and diagnosed with surgical resection of nodules. The lesions on vertebrae thought to be operational scar can be primary focus. However, this focus makes a diagnosis problematic. The lung lesion of the patient turned out to be a Ewing syndrome according to pathological inspection and it has been deemed appropriate to start treatment. After treatment, the lesions on vertebrae shown no change but lung lesions have reduced by 80%, which indicates that the primary focus is the lung. Since the first description of ES and extraskeletal Ewing sarcoma by James Ewing and Tefft et al. these two conditions have been confused histologically with other small cell tumors, including PNETs [8], but recent immunoperoxidase and cytogenic studies indicated that PNETs and ES are the same entity showing varying degrees of neuroectodermal differentiation, and they were categorized into a group known as the Ewing family of tumors [9]. The ES/PNETs family is an uncommon malignant neoplasm and shares common histological features of closely packed small primitive round cells. It most often arises in the soft tissues and bones but has rarely been reported in other sites. Ewing sarcoma is a malignant bone tumor derived from mesenchymal cells of bones, the origin of which is not precisely known [9]. Due to the fact that they have common histopathological and cytogenetical features, Ewing sarcoma is also evaluated within the primitive neuroectodermal tumor (PNET) group [10, 11]. The morphological and immunohistochemical features of pulmonary PNETs are not different from its counterparts of other origins, special diagnostic considerations of other tumors with small, blue, round cell morphology, and primarily small cell carcinoma of the lung is necessary [4]. Lung carcinoids share a similar morphology to PNETs and form rosettes. However, the detection of chromogranin, synaptophysin, and neuron specific enolase is necessary to confirm the diagnosis of lung carcinoid. In addition, differential diagnosis is necessary to distinguish PNETs from metastasis of small cell carcinomas of other origins. Another important diagnostic consideration for both children and adult patients is pulmonary rhabdomyosarcoma, which is also a rare tumor and belongs to the group of small, blue, round cell tumors. Muscle-specific markers such as desmin, myogenin, or myo-D1, characteristic of rhabdomyosarcoma, should be included in the immunohistochemical study [12]. ES is most commonly observed in males and between the ages of 15 and 25 (M/F: 1.1/1) [13]. ES is an aggressive tumor that is most commonly seen in patients under the age of 20 [14]. In a study [15] conducted in Turkey, the median age was 27 years and 19% of all cases were over 30 years of age [15], which correlated with the center's other observations [16, 17]. It is most commonly seen in the lower extremities and the pelvis, and this location comprises about 60% of all cases [18]. Patients mostly complain of pain. Our case was over 32 years of age and no lesions were observed at the lower extremities. The signs and findings at the time of diagnosis are both constitutional and related to the disease site. The first symptoms are in general pain and/or swelling at the primary tumor region. Of the patients who had been treated with the diagnosis of ES at the Mayo clinic, 96% had been reported to have pain, 61% had a palpable mass, and 16% of the patients had pathological fractures [19]. The disease may present with signs from the metastatic sites. Back pain may be the first sign of cord compression secondary to primary or metastatic vertebral tumor and requires immediate evaluation and intervention before permanent neurological damage develops.

In our case, although the patient had vertebral lesions, he did not complain of back pain. History of an operation following a fall from height 2 years ago may be related to the primary lesion, but data from the patient files could not be obtained. Ewing sarcoma is a vascular tumor that metastasizes to the lungs. For our patient, bone lesions are assumed to be operation-related and lung lesions are evaluated as primary lesions. Findings related to the tumor may not be observed on conventional X-rays. However, when present, it is observed as a nonspecific mass, the size of which may vary a wide range. Detection of* EWSR1* gene translocation or amplification is the most reliable marker of PNETs, including those of pulmonary origin [4]. We did not perform this analysis because of our laboratory conditions and this was our shortcoming.

In our case, although the lesions (operation-related lesions) were also present in bones other than the lungs, biopsy was obtained from the largest lesions in the lungs, because the locations of bone lesions were not suitable for diagnostic resection. The lesions on vertebrae detected by PET scan originally thought to be related to the operation can be assumed to be Ewing sarcoma focus; however, after chemotherapy the lung lesions have shown an almost complete regression but vertebrae lesions did not change in size and detectability, which may indicate that the primary focus is not the bone. Ewing sarcoma is a malignancy that is sensitive to chemotherapy. Systemic chemotherapy is effective in reducing the local tumor size besides being effective on the microscopic and macroscopic metastasis. The treatment outcomes of patients who have metastasis are worse than patients who do not. The disease responded well to chemotherapy in our patient in spite of the diffuse lung involvement.

In conclusion, ES is generally considered a childhood disease, but it can also be seen in adults. Thus, early diagnosis and effective treatment carry great importance. Our ES case diagnosed with surgical removal of the nodule had responded well to the treatment.
